# Expert Consensus on the Nutrition Care Process in Guatemalan Hospitals: Findings from a Delphi Study of nutritionDay 2022 Participants

**DOI:** 10.3390/nu17193110

**Published:** 2025-09-30

**Authors:** Karen Girón, Isabel Chinchilla, Christa Gómez, Marietta Lau, María René Oroxon, Elena Díaz, Jorge Gramajo, Abraham Monroy, Arlen Carrera, Massiel Roldán, Cristina Nárez, Ana Verónica Monterroso, María Victoria González, Evelyn Frias-Toral, Jaen Cagua-Ordoñez, Claudia Maza Moscoso, Daniel Simancas-Racines

**Affiliations:** 1 Hospital de Chiquimula, Chiquimula 20001, Guatemala; kargivi11@gmail.com; 2 Facultad de Ciencias de la Salud, Universidad Panamericana de Guatemala, Guatemala City 01010, Guatemala; iecha86@gmail.com; 3 Hospital General San Juan de Dios, Guatemala City 01001, Guatemala; chrisis9@gmail.com (C.G.); mariettalau@gmail.com (M.L.); 4 Hospital Departamental de Totonicapán, Totonicapán 08001, Guatemala; maroroxon@gmail.com; 5 Hospital Privado Quetzaltenango S.A., Quetzaltenango 09001, Guatemala; elenamariadiazruiz@gmail.com; 6 Hospital Regional de Occidente, Quetzaltenango 09001, Guatemala; joregra@yahoo.com; 7 Facultad de Medicina, Universidad de San Carlos de Guatemala, Guatemala City 01001, Guatemala; monroy95abraham@gmail.com; 8 Hospital Roosevelt, Guatemala City 01001, Guatemala; arandere@gmail.com (A.C.); mroldanrodas@gmail.com (M.R.); 9 Corporación de Hospitales, Guatemala City 01001, Guatemala; nareznutricion@gmail.com; 10 Division of Education and Research, Centro Médico Militar, Guatemala City 01001, Guatemala; avmm.27@gmail.com (A.V.M.); congresonutrimilitar@yahoo.com (M.V.G.); 11 School of Medicine, Universidad Espíritu Santo, Samborondón 0901952, Ecuador; efriast@uees.edu.ec; 12 Division of Research, Texas State University, San Marcos, TX 78666, USA; 13 Center for Public Health and Clinical Epidemiology Research (CISPEC), Facultad de Ciencias de la Salud Eugenio Espejo, Universidad UTE, Quito 170527, Ecuador; jean.cagua@ute.edu.ec

**Keywords:** hospital malnutrition, clinical nutrition, nutritional care process, Delphi method, Guatemala, nutritionDay, consensus

## Abstract

**Background:** Disease-related malnutrition (DRM) remains an underdiagnosed condition in Latin American hospitals, with substantial clinical and economic consequences. The global nutritionDay initiative, promoted by ESPEN, provides a standardized audit to evaluate and improve hospital nutritional care. This study aimed to develop expert consensus recommendations to optimize the nutritional care process in Guatemalan hospitals, based on the findings from nutritionDay 2022. **Methods:** A modified Delphi study was conducted, including three meetings held before and after each round to discuss the results. Sixteen clinical nutrition professionals from eleven hospitals participated. A total of 89 items were assessed, with a predefined consensus threshold of 70%. Data were analyzed using R software (version 4.5.0) and Kendall’s W coefficient was applied to evaluate inter-round agreement. **Results:** Consensus was achieved for 51 key recommendations covering nutritional screening, clinical assessment, anthropometry, body composition, functional assessment, biochemical monitoring, dietary intervention, and post-discharge follow-up. The proposed actions are aligned with international guidelines (ESPEN, ASPEN, GLIM) and adapted to the Guatemalan healthcare context. **Conclusions:** This consensus provides a comprehensive and context-specific framework for standardizing and improving hospital nutritional care in Guatemala and similar settings. Its implementation could help reduce DRM prevalence and foster the development of quality indicators and digital tools for clinical nutrition management.

## 1. Introduction

Disease-related malnutrition (DRM) is a significant clinical problem that remains persistently underdiagnosed in hospital settings [1]. Systematic reviews conducted in Latin American hospitals estimate its prevalence to range between 40% and 60%, with substantial adverse consequences such as increased mortality, higher incidence of healthcare-associated infections, and prolonged hospital stays [2]. This burden is further exacerbated by the coexistence of the double burden of malnutrition—undernutrition and overweight—well documented across the region [3,4]. Despite this, multicenter audits have shown that fewer than half of tertiary care hospitals have formalized nutritional care teams and protocols in place, revealing a considerable gap between clinical needs and actual practice [5].

In this context, the international nutritionDay (nDay) initiative—led by the European Society for Clinical Nutrition and Metabolism (ESPEN) and coordinated by the Medical University of Vienna—has become a standardized global audit tool for evaluating the quality of nutritional care in healthcare institutions [6,7,8]. Since its inception in 2006, it has collected data from more than 235,000 hospitalized patients in 74 countries, showing that approximately 45% experience recent weight loss and nearly 50% consume less food than is served to them. Guatemala has been formally participating since 2019, and the 2022 national report documented marked heterogeneity in nutritional screening, assessment, and follow-up processes among participating institutions.

The need to harmonize hospital nutritional care in the country is supported by international position statements such as the Cartagena Declaration (2019), which recognizes nutritional care as a fundamental component of the right to health [9]; the Vienna Declaration (2022), which emphasizes the ethical and clinical value of equitable access to nutritional therapy [10]; and the Asunción Commitment (2023), which calls for the implementation of concrete actions to ensure adequate, timely, and effective nutrition during hospitalization.

Based on the findings from nDay Guatemala 2022, a network of clinical and academic professionals was established. Through a three-round modified Delphi process—with meetings held before and after each round to discuss the results—this group developed a set of 51 structured recommendations covering all stages of the hospital nutrition care process, from initial screening to post-discharge follow-up. This article describes the methodology used and the results obtained, with the aim of: (i) providing a technical framework adapted to the Guatemalan hospital context and other similar settings, (ii) strengthening the implementation of comprehensive quality management systems in clinical nutrition, and (iii) promoting future evaluation of the clinical, economic, and organizational impact of these recommendations in practice.

## 2. Materials and Methods

### 2.1. Study Design

A modified Delphi consensus study was conducted over three successive rounds. This design enabled the structured integration of expert opinions on key practices of the hospital Nutrition Care Process (NCP) in Guatemala, drawing on the experience of professionals responsible for the national implementation of nDay 2022. The methodology followed the recommendations of the JBI Manual for Evidence Synthesis (2024), as well as internationally recognized standards for developing clinical practice guidelines, including AGREE-II and RIGHT.

The process combined the structured quantification of the Delphi methodology (questionnaires with predefined response options) with qualitative deliberation through pre- and post-round discussion meetings, which allowed for synchronous validation and iterative refinement of items. This was a consensus-based, prospective study without direct clinical intervention or patient-level data collection.

### 2.2. Panel Selection

The expert panel was selected through purposive sampling. Sixteen professionals responsible for implementing nDay 2022 in eleven Guatemalan hospitals (public, private, and Ministry of Defense) were invited to participate. Inclusion criteria were: (1) at least two years of experience in hospital clinical nutrition, and (2) direct involvement in nDay data collection. Panelists also had to be actively engaged in inpatient nutritional care and represent diverse institutional contexts.

Panelists agreed in principle to complete the three Delphi rounds; however, participation remained voluntary. To maximize retention, individualized invitations, two automated reminders per round, and fixed deadlines (two weeks per round) were implemented. Losses in later rounds were exclusively due to logistical issues (e.g., workload, schedule conflicts) and not to dissent.

The final panel consisted of 15 clinical nutritionists and one physician, with 93.8% holding direct patient care responsibilities. The inclusion of the physician was justified by his institutional leadership role in coordinating nutrition-related practices and contribution of clinical expertise. All participants signed the ICMJE authorship declaration and completed a conflict-of-interest form, with no relevant disclosures reported.

### 2.3. Delphi Process Structure

**Figure 1:**
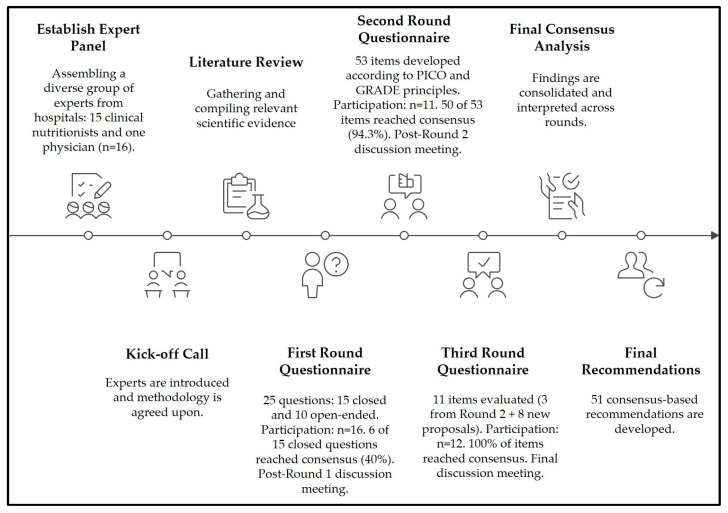
Delphi consensus process for developing recommendations in hospital nutrition care in Guatemala. The figure depicts the sequential stages of the modified Delphi methodology, beginning with expert panel establishment and progressing through three iterative rounds of evaluation. Each round includes the number of items assessed and the proportion of consensus achieved, with post-round discussion meetings incorporated to refine and validate recommendations. The process was structured according to internationally recognized methodological principles (PICO and GRADE) and validated through stability analysis using Kendall’s W coefficient. This iterative approach guided the development of 51 consensus-based recommendations for hospital nutrition care.

The Delphi process consisted of three iterative rounds (Figure 1). Round 1 included a mixed questionnaire of 25 items: 15 multiple-choice closed questions with predefined answers and 10 open-ended questions aimed at describing institutional practices in nutritional screening, clinical assessment, dietary intervention, and monitoring. The closed questions were designed by the methodological team, which comprised three researchers with expertise in clinical nutrition and evidence synthesis and were validated through independent expert review. A pilot test was conducted with a subgroup of three panelists to ensure clarity, feasibility, and completion time, leading to minor adjustments before launch.

The results from this round identified gaps and divergences, which were then used to inform the development of subsequent rounds. Round 2 consisted of 53 Likert-type statements (1–5), structured according to the PICO framework, grouped by NCP domains, and prioritized using GRADE criteria. These statements were derived both from the findings of Round 1 and from international clinical guidelines.

Round 3 involved the reassessment of three items that had not achieved consensus in the previous round, together with eight new items proposed by the panel during Round 2 discussions, for a total of 11 statements. The inclusion criteria for Round 3 items were strictly defined: only items without prior consensus and those newly proposed by panelists were retained. These additional items addressed previously unexplored components such as post-discharge nutritional follow-up and the systematization of clinical–nutritional indicators.

All questionnaires were developed in Google Forms, reviewed by an independent subgroup of three panel members, and pilot-tested for clarity, content relevance, and completion time. Each round remained open for four weeks, with an automated reminder sent on day 21 to maximize response rates. Participation included 16 experts in Round 1, 11 in Round 2, and 12 in Round 3, the latter explained by the re-engagement of one panelist who had been unable to respond in Round 2 due to logistical reasons. To ensure transparency, results are reported as both absolute counts and percentages (100% [16/16], 68.8% [11/16], and 75% [12/16], respectively). No imputation of missing responses was performed; only valid answers were analyzed. To enhance transparency, post-round meetings were held to discuss general aspects of the findings and to guide the design of subsequent questionnaires; however, these meetings did not alter the responses already submitted.

### 2.4. Consensus Criteria

Consensus for each item was defined a priori as at least 70% of participants selecting categories 4 (“agree”) or 5 (“strongly agree”) on the Likert scale. This threshold has been recommended as appropriate for Delphi studies in public health, according to the JBI Manual for Evidence Synthesis and recent systematic reviews.

Given the relatively small panel size (n = 16), results are presented in both absolute numbers and percentages to ensure transparency. In practice, the 70% threshold corresponded to a minimum of 12/16 participants in Round 1, 8/11 in Round 2, and 9/12 in Round 3. Items that did not reach this level of agreement were either excluded or, when deemed of clinical importance, re-evaluated in subsequent rounds based on panel discussion and reformulation. The reduction in participation by Round 2 and Round 3 is acknowledged as a limitation that may have influenced consensus stability, although iterative validation and the use of Kendall’s W coefficient were applied to mitigate this risk.

### 2.5. Data Processing

Files were exported in .xlsx format and processed in R version 4.5.0. Variable names were standardized using the janitor: clean_names() function. Responses were numerically recoded on a uniform 5-point Likert scale, where “strongly disagree” = 1 and “strongly agree” = 5. Entries such as “NA”, “N/A”, or “not applicable” were treated as missing values, and no imputation was performed; all analyses were conducted exclusively with complete responses.

For Round 3, question headers were harmonized using a correspondence dictionary developed by the methodological team, ensuring longitudinal comparability with Round 2. This allowed for consistent tracking of items across rounds, particularly for those that were reformulated or newly introduced.

### 2.6. Statistical Analysis

The percentage of agreement was calculated for each item and round as the proportion of participants selecting categories 4 (“agree”) or 5 (“strongly agree”) on the Likert scale. To ensure transparency in a small panel (n = 16), both absolute counts and percentages are reported.

To assess the stability of consensus across rounds, Kendall’s W coefficient of concordance was applied to the three items common to Rounds 2 and 3. Values of Kendall’s W range from 0 (no agreement) to 1 (perfect agreement), providing a quantitative measure of group consistency and the direction of change in ratings over time. Items newly introduced in Round 3 were analyzed exclusively in a cross-sectional manner, using absolute and relative frequencies. No imputation was performed for missing responses.

All statistical analyses were conducted in R (version 4.5.0) using the tidyverse for data wrangling and the irr package for agreement statistics.

### 2.7. Reproducibility and Availability

All processing and analyses were performed in R (version 4.5.0) using the packages tidyverse, janitor, irr, and writexl. The complete scripts are provided separately for each Delphi round (Round 1, Round 2, Round 3) and for the Kendall’s W stability analysis. These materials, along with Supplementary Materials outputs, are available in the Mendeley Data repository (10.17632/3tkbc7d3pn.1) under an open-access license for verification and reuse.

## 3. Results

### 3.1. Characteristics of the Expert Panel

The panel consisted of 16 professionals—15 clinical dietitians and one physician—responsible for the implementation of nDay 2022 in their institutions. Most participants (93.8%) were engaged in hospital clinical nutrition practice; 81.3% worked in public hospitals, 12.5% in private institutions, and 6.2% in Ministry of Defense facilities. Additionally, 12.5% reported complementary activities in teaching or research.

### 3.2. Mapping of Clinical Practices and Emerging Consensus Lines on Hospital Nutritional Care: Findings from Round 1

#### 3.2.1. Frequency Analysis and Consensus in Closed Questions

Among the multiple-choice items, six reached the predefined consensus threshold (≥70%). Seventy-five percent of participants reported having institutional protocols for nutritional assessment and treatment, and 87.5% indicated that they regularly performed periodic evaluations of food intake. Consensus was also achieved regarding the use of body mass index (93.8%) and body composition assessment (81.3%) as routine indicators. Regarding interventions, 87.5% prioritized optimizing oral diets, and a similar proportion reported informing patients about their nutritional status at hospital discharge.

In contrast, other questions revealed highly variable clinical practices. The most frequently mentioned screening tool was the Nutritional Risk Screening (NRS) (43.8%), with no consensus on its universal applicability. Differences were also observed in the frequency of anthropometric, biochemical, and clinical monitoring, as well as in strategies for outpatient follow-up.

#### 3.2.2. Emerging Categories from Open-Ended Responses

The thematic analysis of open-ended questions reinforced and contextualized the quantitative findings. Considerable heterogeneity was observed in the updating of institutional protocols (intervals ranging from 1 to 5 years) and in the clinical sources used when formal guidelines were absent. Food acceptability assessments were reported as sporadic and mainly based on structured questionnaires, whereas objective methods such as photographic recording of meal trays were rarely mentioned.

The main barriers to oral intake included poor palatability and presentation of meals, patients’ clinical conditions, and the cultural inadequacy of hospital diets. In response, participants proposed improving food presentation and variety, personalizing meal preparations, and strengthening patient support from healthcare staff. To prevent unintentional weight loss, participants suggested enhancing early screening, improving diet quality, and consolidating multidisciplinary care teams.

### 3.3. Expert Consensus on the Nutritional Care Process in Guatemalan Hospitals: Findings from Round 2

Of the 53 items evaluated, 50 (94.3%) reached the consensus threshold, reflecting a high level of agreement among experts regarding multiple components of nutritional care. These included diagnostic criteria, therapeutic strategies, assessment procedures, and discharge practices. In contrast, three items (5.7%) did not achieve consensus, highlighting areas where differences in opinion or barriers to practical implementation persist.

The items with the strongest consensus included the systematic incorporation of a detailed physical examination into nutritional assessment (100%), the use of percentage weight loss as a clinical indicator (100%), and the adoption of validated tools to estimate weight and height in hospitalized patients (≥90%). High agreement was also reached on the need to inform patients at discharge about their nutritional status and to document this information in the medical record (≥93.8%).

Regarding nutritional diagnosis, participants largely supported the use of the GLIM criteria and the application of the NRS in adults under 65 years, as well as the MNA in older adults. The importance of implementing updated institutional protocols, assessing daily intake, and considering hospital diet acceptability as part of routine clinical monitoring was also underscored. In terms of intervention, experts prioritized optimized oral diets as the first-line therapeutic strategy, followed by individualized nutritional support tailored to the patient’s clinical condition.

The few items that did not reach consensus were mainly related to operational or contextual aspects, such as the ideal frequency of biochemical and anthropometric monitoring, the use of non-standardized tools to evaluate food acceptability, and specific mechanisms to ensure post-discharge nutritional follow-up in settings with structural limitations.

### 3.4. Final Validation of Consensus on Hospital Nutritional Care Practices: Findings from Round 3

Twelve experts reviewed eleven statements (three reevaluated and eight new). All reached the consensus threshold (83.3–100%). Among the most strongly supported recommendations was the use of the NRS as the preferred nutritional screening tool for adults under 65 years, and the use of the MNA in older adults—both of which had already been discussed and endorsed in Round 2. Consensus was also confirmed regarding the individualized use of BMI values between 22 and 25 kg/m^2^ as a clinical approximation to ideal body weight, alongside recognition of the percentage of weight loss as a clinically valuable indicator of nutritional risk. These recommendations were regarded as consistent with evidence-based international practices and their applicability across hospital settings with variable resource availability.

Practical measures were also validated, including the use of ulna length to estimate stature when other anthropometric techniques are not feasible, and the use of handgrip strength as a functional proxy in the absence of dynamometry. Regarding dietary management, consensus was achieved on the appropriateness of employing multiple complementary methods for intake assessment, including 24 h recalls, evaluation of usual diet, and plate photographs. For follow-up, experts reaffirmed the need for weekly anthropometric and biochemical monitoring, particularly in patients receiving nutritional support.

### 3.5. Sensitivity Analysis

The Kendall’s W coefficient of concordance obtained for the three items evaluated across both Rounds 2 and 3 was 0.073, indicating low initial agreement among the experts. This finding reflects the active deliberation of the panel regarding initially controversial statements and highlights the iterative and reflective nature of the Delphi method, which enables the transformation of initial disagreement into informed consensus through successive rounds of structured evaluation.

### 3.6. Final Consensus Recommendations on the Hospital Nutritional Care Process in Guatemala

At the conclusion of the Delphi process, a comprehensive set of clinical recommendations was established, reflecting expert consensus on hospital nutritional care practices in Guatemala. These recommendations encompass the full continuum of the nutritional care process, from risk identification to long-term follow-up, and were informed by both scientific evidence and the pragmatic expertise of professionals across national institutions.

The consolidated framework emphasizes the systematic implementation of nutritional screening at hospital admission, followed by an integrated clinical assessment that incorporates objective measures, medical history, and drug–nutrient interactions. Physical examination and anthropometric assessment are standardized as core practices, complemented by body composition analysis—notably bioelectrical impedance and phase angle—as prognostic markers. Functional evaluation, primarily through handgrip strength, is recognized as an essential component of comprehensive nutritional status assessment.

**Figure 2:**
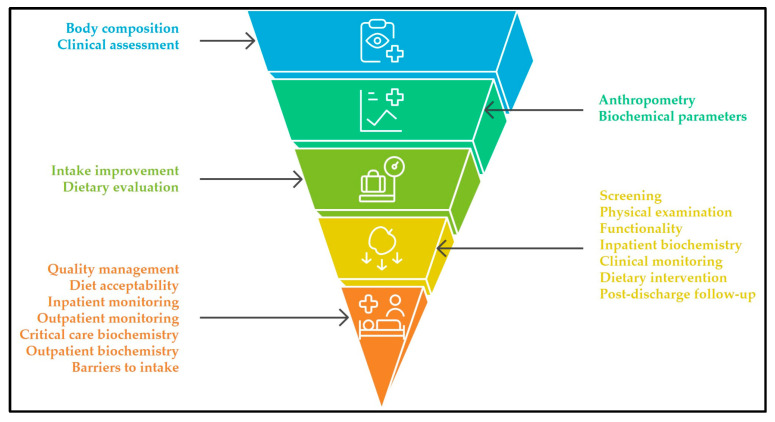
Consensus-based pyramid of hospital nutritional care recommendations in Guatemala. The base represents domains with the largest number of recommendations (e.g., screening, monitoring, dietary practices), while the upper tiers contain fewer, more specialized practices (e.g., body composition, advanced assessment), reflecting a gradient from foundational to advanced strategies.

A strong focus is also placed on biochemical monitoring protocols, differentiated by clinical context (inpatient, outpatient, critical care, and parenteral nutrition). Likewise, strategies addressing diet quality, acceptability, and intake optimization were prioritized, along with the systematic documentation of barriers to intake. The framework further recommends multidisciplinary monitoring, extending beyond hospitalization to ensure structured post-discharge follow-up at defined intervals. The hierarchical distribution of these recommendations is visually synthesized in Figure 2.

**Figure 3:**
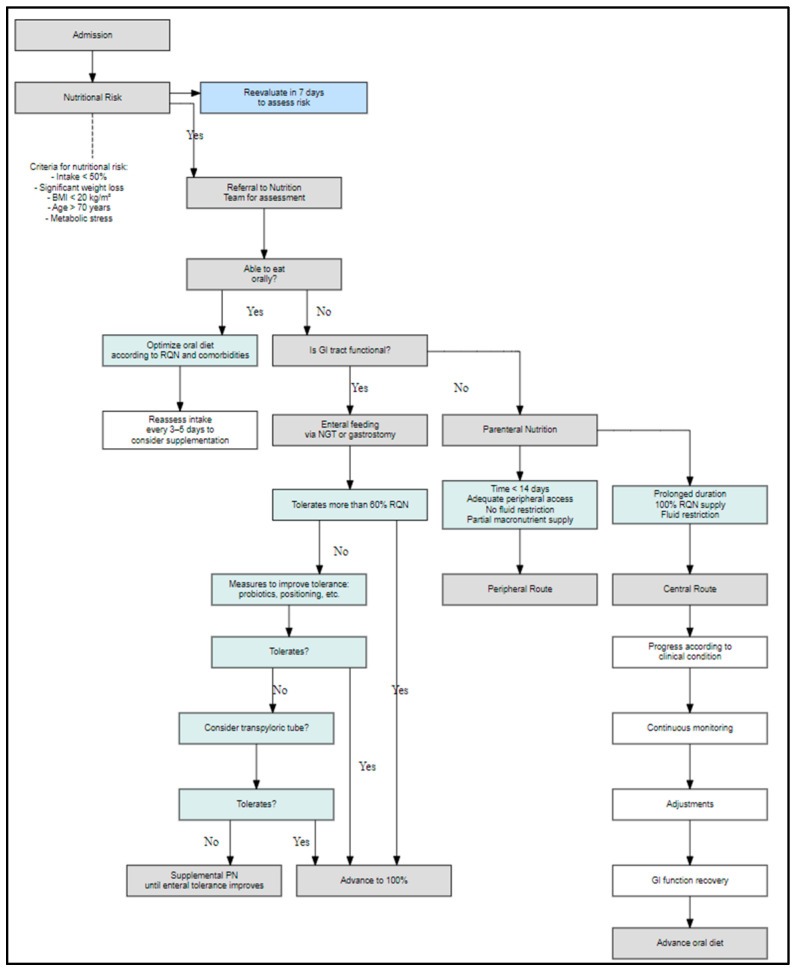
Algorithm for nutritional support decision-making in hospitalized patients according to nutritional risk assessment. Clinical decision flowchart used to guide the type of nutritional support in hospitalized patients based on the initial nutritional risk evaluation. The algorithm considers oral feeding capacity, gastrointestinal functionality, and tolerance to enteral nutrition to determine the most appropriate route, including parenteral nutrition options when necessary.

Additionally, the expert panel validated the integration of structured decision-making algorithms into hospital protocols. This is reflected in Figure 3, which operationalizes one of the recommendations included in Table A1, illustrating the clinical decision pathway for oral, enteral, or parenteral nutritional therapy based on gastrointestinal functionality and patient condition.

The complete set of recommendations, organized by domain, is detailed in Table A1 (Appendix A). This table provides a practical reference for clinicians and policymakers to standardize hospital nutritional care, while the material supplement offers quantitative data on the level of agreement reached across Delphi rounds.

## 4. Discussion

This national consensus articulates 51 recommendations covering the continuum of hospital nutritional care, and their comparison with international guidelines demonstrates substantial convergence with ESPEN, ASPEN, and GLIM standards, while also providing concrete operationalization for clinical practice in health systems with heterogeneous resources. In this regard, the document does not merely reiterate international principles but adapts them to a framework of immediate applicability within the Guatemalan hospital context.

First, the decision to standardize the NRS-2002 in adults under 65 years and the MNA in adults over 65 years is aligned with the requirement for systematic screening within the first 24–48 h of admission, as established by ESPEN and ASPEN, thus ensuring early detection and interinstitutional comparability [11,12,13,14]. Moreover, the two-step model proposed by GLIM (screening followed by diagnostic evaluation) supports the choice of age-specific tools, reducing methodological variability without departing from global consensus [12]. Taken together, this local strategy does not contradict the international framework but rather operationalizes it, facilitating implementation and audit in hospitals with diverse capacities.

Complementarily, the recommendation to perform an objective nutritional evaluation at admission, combining the SGA with objective measures such as muscle mass and biochemical parameters, reflects the phenotypic–etiologic logic of the GLIM framework and the multidimensional vision of ESPEN/ASPEN to confirm malnutrition and guide intervention [12]. It is important to highlight, however, that GLIM does not include serum proteins as diagnostic criteria due to their nonspecificity in inflammatory contexts, shifting the emphasis toward inflammatory burden as the etiologic axis [12,15]. This nuance is essential to avoid misclassification in patients with systemic inflammatory response. At the same time, integrating medical history, comorbidities, drug–nutrient interactions, and lifestyle habits broadens the perspective to a biopsychosocial approach consistent with ESPEN’s geriatrics recommendations [16].

Regarding physical examination and anthropometry, the inclusion of a systems-based nutritional physical exam aimed at detecting loss of muscle, adipose tissue, and micronutrient deficiency signs is consistent with the NFPE practice recommended by the Academy/ASPEN, as well as with the use of SGA and GLIM as reference frameworks [17]. Similarly, the proposal to employ alternative methods to estimate height, such as ulna length or knee height, and the cautious interpretation of BMI—emphasizing weight loss percentage and central adiposity—reflect the growing body of evidence that questions the isolated value of BMI, particularly in older adults [18]. The mandate to validate BMI only when combined with body composition indicators reinforces the international emphasis on multidimensional evaluation and provides operational clarity to a frequently debated issue.

In terms of body composition and functionality, the recommendation to employ BIA and interpret the phase angle is in line with studies demonstrating their prognostic value for mortality and hospital length of stay, even though international guidelines avoid universal cut-offs and advise using these parameters as complementary indicators [19,20]. Likewise, the incorporation of calf circumference and handgrip strength as simple, reproducible tools is consistent with EWGSOP2 and AWGS criteria as well as with ESPEN’s positions, thereby democratizing functional evaluation in settings with limited technology [21,22,23,24].

A similar logic of pragmatism applies to monitoring. While international guidelines generally recommend flexible intervals, the Guatemalan consensus introduces explicit frequencies—24–48 h in ICU, weekly for enteral or oral support, 24–72 h for parenteral nutrition, and 1–3 months for outpatient follow-up—which facilitates standardization and traceability in clinical practice [25]. This proposal is complemented by the definition of disease-specific laboratory panels (renal, hepatic, dyslipidemia, parenteral nutrition, and general inpatient evaluation), which translate international recommendations into concrete laboratory checklists, enhancing patient safety and optimizing diagnostic resource allocation [26,27]. However, such prescriptive detail requires clinical flexibility to avoid both overuse in stable patients and under-monitoring in unstable scenarios.

Nutritional intervention strategies also show coherence with the international canon. The principle that the oral or enteral route must be prioritized whenever the gastrointestinal tract is functional is firmly established by ESPEN, ASPEN, and NICE, and its representation through a clinical decision algorithm strengthens consistency in care. Similarly, the recognition of multifactorial barriers to intake and the use of 5-point hedonic scales to assess diet acceptability resonate with evidence linking organoleptic quality and patient experience to adherence and adequate energy intake [16,25,28]. The cautious indication of orexigenic agents only when reversible causes have been excluded is also consistent with international guidelines acknowledging their limited benefit and potential adverse effects [29].

The evaluation of food intake is likewise consistent with best international practices, recommending the registration of habitual diet at admission, the use of 24-h recalls in clinically stable patients, and, when feasible, the application of photographic methods as a practical way to quantify consumption [30,31,32]. Finally, the indication to establish a structured post-discharge follow-up at least every three months is supported by ASPEN’s MQii toolkit and meta-analyses showing reductions in mortality and readmissions when nutritional support is continued after hospitalization [33].

Taken together, the 51 recommendations of the Guatemalan consensus not only reproduce the core of ESPEN, ASPEN, and GLIM, but also enrich it by providing operational details such as minimum monitoring intervals, disease-specific laboratory panels, and decision algorithms. These contributions translate general principles into applicable and auditable instruments, which may accelerate implementation in heterogeneous health systems. Nonetheless, predictable tensions persist: the need to differentiate between diagnostic and follow-up use of biochemical parameters, the advisability of adjusting rigid intervals according to clinical risk, the validation of anthropometric cut-offs in Latin American populations, and the cautious interpretation of phase angle in the absence of universal thresholds.

Moreover, full implementation faces structural limitations. Several recommendations rely on moderate or extrapolated evidence from non-Latin American contexts, and the uneven availability of tools such as BIA, dynamometers, or biochemical resources may condition immediate applicability. In this scenario, a staggered implementation strategy—distinguishing between an essential package (early screening, SGA plus objective measures, calf circumference, handgrip strength, basic algorithms) and an advanced package (BIA, phase angle, hedonic scales, differentiated laboratory panels, systematic audits)—appears to be the most realistic pathway to ensure progressive and sustainable adoption.

Finally, these measures should be accompanied by quality indicators and digital tools enabling real-time monitoring. Key indicators include the percentage of patients screened within 48 h, documentation of handgrip and calf circumference, adequacy of caloric–protein provision, adherence to laboratory monitoring windows according to risk level, existence of post-discharge follow-up plans, and patient satisfaction with hospital diet. In addition, the future research agenda should prioritize multicenter pragmatic trials in Latin America evaluating the impact of these recommendations on mortality, readmissions, hospital stay, and costs, as well as the calibration of anthropometric and functional cut-offs for regional populations. A methodological limitation to acknowledge is the variation in panel participation across rounds (16 in Round 1, 11 in Round 2, and 12 in Round 3). The slight increase in Round 3 was due to the re-engagement of a panelist who could not participate in Round 2 for logistical reasons. While this does not undermine the validity of the process, it may have influenced the stability of consensus in items reassessed during Round 3.

## 5. Conclusions

This multidisciplinary consensus synthesizes 51 prioritized recommendations for hospital nutritional care, developed through a structured Delphi process and adapted to the context of health systems in Latin America. The result is a technical–operational framework that spans from screening to post-discharge follow-up, incorporating key elements such as functionality, dietary acceptability, and targeted monitoring according to the type of nutritional support and comorbidity.

Unlike fragmented guidelines or those focused primarily on high-income regions, this set of recommendations offers an integrated and applicable perspective in resource-limited settings, fostering cost-effective, replicable, and patient-centered interventions. Their progressive adoption may contribute to reducing the burden of hospital malnutrition, optimizing the use of clinical resources, and standardizing the quality of nutritional care.

This work provides a foundation for the development of performance indicators, digital clinical support tools, and future implementation research aimed at validating their impact on clinical and economic outcomes in hospitals across the region.

## Data Availability

The data supporting the findings of this study are available upon reasonable request from the corresponding author. Restrictions apply to the availability of these data due to institutional confidentiality agreements.

## Supplementary Materials

The following supporting information can be downloaded at: https://www.mdpi.com/article/10.3390/nu17193110/s1, Supplementary Analysis S1: Analysis of open-ended questions from Round 1 of the Delphi consensus on hospital nutrition care in Guatemala; Table S1: Consolidated results of the 15 multiple-choice questions included in Round 1 of the Delphi study; Table S2: Results of Likert-type questions from Round 2 of the Delphi consensus on hospital nutritional care in Guatemala; Table S3: Results of Likert-type questions from Round 3 of the Delphi consensus on hospital nutritional care in Guatemala; Table S4: Alignment of Delphi round questions with final recommendations for hospital nutrition care in Guatemala.

## Abbreviations

The following abbreviations are used in this manuscript:

BMI	Body Mass Index (Índice de Masa Corporal)
GLIM	Global Leadership Initiative on Malnutrition
IMC	Índice de Masa Corporal
MNA	Mini Nutritional Assessment
NRS	Nutritional Risk Screening
PCN	Proceso de Cuidado Nutricional
nDay	nutritionDay
FAO	Food and Agriculture Organization
OMS	Organización Mundial de la Salud
MSPAS	Ministerio de Salud Pública y Asistencia Social
USAC	Universidad de San Carlos de Guatemala
UNESCO	United Nations Educational, Scientific and Cultural Organization

## Appendix A

**Table A1:** Final expert consensus recommendations on the hospital nutritional care process in Guatemala.

General Domain	Recommendation
Screening	Use the NRS in adults under 65 years and the MNA in adults over 65 years as standardized nutritional screening tools.
Clinical assessment	Conduct an objective nutritional assessment for every hospitalized patient upon admission. Complement the Subjective Global Assessment (SGA) with objective measurements to achieve a comprehensive nutritional evaluation. When applying GLIM criteria, include objective measures such as muscle mass and biochemical parameters. Incorporate relevant clinical history and medical diagnoses as part of the nutritional evaluation. Investigate and document potential drug–nutrient interactions during the clinical assessment. Systematically inquire about lifestyle habits with possible nutritional impact: alcohol, tobacco, drug use, physical activity, and contraceptives.
Physical examination	Include a systems-based review as part of the nutritional physical examination. Routinely assess muscle mass, adipose tissue, and clinical signs of micronutrient deficiencies.
Anthropometry	Measure height using a stadiometer, arm span, or knee height; use ulna length when other methods are unavailable. Estimate ideal weight using a reference BMI of 22–25 kg/m^2^, adjusted to the patient’s clinical condition. Consider physiological variations in BMI in adults over 65 years for adequate interpretation. Interpret percentage weight loss as a clinical indicator of nutritional risk. Validate the use of BMI only when combined with body composition and central adiposity measures.
Body composition	Use bioelectrical impedance analysis (BIA) in hospitalized patients as a feasible tool for body composition assessment. Interpret the phase angle derived from BIA as a nutritional prognostic parameter. Recognize the utility of clinical anthropometry as a complementary method in body composition assessment. Standardize procedures for anthropometric measurements in the hospital setting. Consider waist circumference cut-offs ≥94 cm in men and ≥80 cm in women as indicators of high risk for chronic noncommunicable diseases. Assess sarcopenia risk in hospitalized adults using clinical or instrumental methods. Measure calf circumference as an indirect indicator of muscle mass, using cut-offs of 34/33 cm (moderate risk) and 32/31 cm (severe risk).
Functionality	Routinely measure muscle strength using handgrip dynamometry. Use handgrip strength as a valid clinical alternative when a dynamometer is not available.
Quality management	Update institutional protocols on hospital nutritional care every 2 to 5 years.
Diet acceptability	Implement 5-point hedonic scales to evaluate hospital diet acceptability.
Inpatient monitoring	Perform weekly anthropometric measurements in hospitalized patients.
Outpatient monitoring	Perform anthropometric measurements in outpatients every 1 to 3 months, depending on nutritional risk.
Critical care biochemistry	Monitor biochemical parameters every 24–48 h in ICU patients.
Inpatient biochemistry	Monitor biochemical parameters at least once per week in patients receiving enteral and/or oral nutritional support. Monitor biochemical parameters every 24–72 h in patients receiving parenteral nutrition.
Outpatient biochemistry	Schedule biochemical monitoring in outpatients every 1 to 3 months.
Biochemical parameters	Establish standardized biochemical panels differentiated by pathology: renal, hepatic, dyslipidemia, parenteral nutrition, and general evaluation. In patients with liver disease: glucose, creatinine, BUN, electrolytes, uric acid, triglycerides, total cholesterol, LDL, HDL, albumin, alkaline phosphatase, AST, ALT, total/direct/indirect bilirubin, and complete blood count. In patients with kidney disease: glucose, creatinine, BUN, electrolytes, uric acid, urine protein and glucose, and complete blood count. In patients with dyslipidemia: glucose, creatinine, BUN, electrolytes, uric acid, triglycerides, total cholesterol, LDL, HDL, albumin, alkaline phosphatase, and complete blood count. In hospitalized patients: glucose, creatinine, BUN, electrolytes, uric acid, triglycerides, total cholesterol, LDL, HDL, albumin, alkaline phosphatase, CRP, and complete blood count. In patients with parenteral nutrition: glucose, creatinine, BUN, electrolytes, triglycerides, total cholesterol, LDL, HDL, albumin, AST, ALT, alkaline phosphatase, total/direct/indirect bilirubin, and complete blood count.
Clinical monitoring	Record daily clinical monitoring as part of nutritional follow-up. Record clinical monitoring at least three times per week in hospitalized patients.
Dietary intervention	Optimize oral diet whenever the gastrointestinal tract is functional. Apply clinical decision-making algorithms for selecting the most appropriate nutritional therapy (Figure 2).
Barriers to intake	Recognize the multifactorial causes of reduced intake, including medical, organoleptic, cultural, and social factors.
Intake improvement	Implement strategies to improve the preparation, presentation, and variety of hospital foods. Ensure multidisciplinary support in monitoring food intake. Consider the use of appetite stimulants in cases of decreased intake without reversible cause.
Dietary evaluation	Record the patient’s usual diet at admission as a baseline for nutritional follow-up. Use the 24 h recall as a valid tool to estimate hospital intake in clinically stable patients. Employ photographic plate methods before and after meals as a practical visual alternative to assess hospital food consumption.
Post-discharge follow-up	Provide structured nutritional follow-up after hospital discharge. Monitor patients’ nutritional status at least every three months during post-discharge follow-up.

Note: Recommendations were developed based on the consensus of national experts who participated in nDay 2022, Guatemala. These practices were validated through a three-round Delphi process, applying a consensus threshold of ≥70% on a Likert-type scale. The recommendations are organized according to the stages of the nutritional care process.
