# Supplementary material for: Expert Consensus on the Nutrition Care Process in Guatemalan Hospitals: Findings from a Delphi Study of nutritionDay 2022 Participants
Source: Nutrients. 2025 Sep 30;17(19):3110. doi: 10.3390/nu17193110 (PMC12525630; doi:10.3390/nu17193110)
Supplement: Supplementary file 1 [file nutrients-17-03110-s001.zip › nutrients-3866145-supplementary.pdf]

Supplementary Analysis S1

Analysis of Open-Ended Questions

Analysis Design:

Open-ended questions were included in the exploratory round (Round 1) of the Delphi study with the aim of capturing qualitative information to complement the closed-ended data. A total of ten open-ended questions (Questions 5, 6, 10, 13, 17, 18, 19, 20, 24, and 25) were identified within the self-administered instrument directed at healthcare professionals involved in hospital nutritional management.

Analysis Procedure:

Each open-ended response was independently reviewed by two researchers with expertise in qualitative analysis. A thematic content analysis approach was applied to identify patterns, emerging categories, and relative frequencies. Responses were first coded manually and subsequently organized into tables by question. Categories were developed inductively, respecting the participants' own wording, while grouping concepts with similar meanings.

Categorization Criteria:

Responses that were blank, coded as "NA," "0," or deemed irrelevant were excluded from the analysis. Answers were preserved in their entirety when they provided rich content or when categorization would result in loss of meaning. In cases where a response contained more than one key concept, multiple coding was applied.

Software:

Database processing and category tabulation were performed using R (version 4.5.0), while final editing of the supplementary material was carried out in Microsoft Word 2021. For editorial review purposes, all categorized responses were consolidated into a single supplementary file.

Results

Question 5

"If the answer is yes, how often do you update them?"
Open-ended response – 16 panelists, 12 valid responses

Normalized Category*	Examples of Original Text	n	% of Respondents†
Annual (≤ 12 months)	"Anual," "Cada año," "Cada año"	5	41.7%
Triennial (≈ 3 years)	"Cada 3 años," "Cada tres o cuatro años"	3	25.0%
Quadrennial (≈ 4 years)	"Cada 4 años"	1	8.3%
Quinquennial (≈ 5 years)	"5 años"	1	8.3%
3–5 years (range)	"Cada 3 a 5 años"	1	8.3%
Contextual comment	"In my positive answer I am referring to what I do in the clinic… Cobán."	1	8.3%
No response / NA / 0	—	5‡	—

* Synonymous responses were grouped ("Anual" ≡ "Cada año").
† Denominator = 12 panelists who provided legible text.
‡ Three "NA," one "0," and one blank field.
Key finding: 42% of the panel update their protocols annually, while another 42% do so every ≥3 years, reflecting substantial heterogeneity.

Question 6

"If the answer is no, what do you rely on to conduct your evaluation and treat your patients?"
(Only for centers that do not have a formal protocol)

Inductive Category*	Example Statements	n	% of Respondents†
Individual academic training	"Varied knowledge acquired during the master's degree"	1	25%
International protocols/guidelines	"International protocols are used"	1	25%
Comprehensive ad hoc clinical evaluation	"Nutritional status, medical diagnosis, medical treatment, biochemistry"	1	25%
Updated guidelines & documents	"Updated guidelines and documents"	1	25%
No response / NA / 0	—	12	—

* Responses were grouped through open reading and consensus between two reviewers.
† Denominator = 4 panelists who provided interpretable text (12/16 left the cell blank, "NA," or "0").
Key finding: Among hospitals without a protocol, reference sources were heterogeneous and evenly distributed across personal training, international guidelines, comprehensive clinical judgment, and updated literature. The low response rate (25%) suggests that most institutions either do have a protocol or chose to omit the question, reinforcing the need to standardize national criteria.

Question 10

"If the answer is yes, how do you measure diet acceptability?"
(16 panelists; 7 provided a specific method)

Methodological Category*	Literal Examples	n	% (of 7)
Survey / questionnaire — satisfaction or hedonic	"Encuestas," "Por encuesta," "Cuestionario hedónico," "Weekly food satisfaction survey," "Questionnaires and interviews"	5	71.4%
Photographic record + daily interview	"Photographic record of the dish before and after eating, daily interview with the patient"	1	14.3%
Measurement limited to nutritionDay	"On nutritionDay"	1	14.3%
No response / NA / 0	—	9†	—

* Grouping induced by two reviewers ($\kappa = 1$).
† Nine panelists left the cell blank, "NA," or "0"; not included in the denominator.
Key finding: The vast majority of those who assess acceptability (5/7) rely on structured surveys (satisfaction or hedonic). More objective methods, such as photographic records of trays, are rarely used (14%), and 14% report measuring only on nutritionDay, suggesting sporadic evaluation practices.

Question 13

"Which biochemical parameters do you most commonly use?"
(16 panelists; multi-component responses)

Biochemical Macro-Category*	Cited Examples	n panelists	% (N = 16)
Lipid profile (total cholesterol, HDL/LDL, triglycerides)	"Lipid profile," "cholesterol and triglycerides"	11	68.7%
Renal tests (creatinine, BUN/urea, "renal function")	"Creatinine, BUN," "renal tests"	10	62.5%
Serum electrolytes (Na, K, Cl, "electrolytes")	"Electrolytes," "sodium and potassium"	10	62.5%

Glucose / glycemia (plasma glucose, HbA1c)	"Glucose," "Glu," "glycosylated hemoglobin"	9	56.2%
Liver tests (transaminases, bilirubin, ALP, "liver function")	"Liver tests," "bilirubin, transaminases"	8	50.0%
Serum proteins (albumin, total proteins)	"Albumin," "total proteins"	6	37.5%
Complete blood count (Hb, Hct, cell count)	"Complete blood count," "Hb"	5	31.2%
Calcium–phosphorus (Ca, P, iPTH)	"Calcium and phosphorus," "Ca, P, iPTH"	3	18.7%
Inflammatory markers (CRP, IL-6)	"CRP," "IL-6"	2	12.5%
Others (CPK, pancreatic enzymes)	"Pancreatic function, CPK"	1	6.2%

* Thematic grouping was conducted by two independent reviewers (κ = 0.92). Percentages allow for multiple responses; a single participant may appear in several categories.

Question 17

"What type of nutrition do you prioritize in your patients when initiating management, and why?"
(16 panelists – multiple responses, thematically consolidated)

Prioritized Macro-Category*	Main Reasoning Cited	n panelists	%
Oral diet as first line	• More physiological • Lower cost • Respects patient habits	11	68.8%
Oral diet + oral nutritional supplements (ONS)	• Hospital diets insufficient • Logistical ease compared to enteral	3	18.8%
Oral → Enteral if requirements not met	• Depends on clinical status and GI functionality	2	12.5%
Enteral / parenteral / mixed nutrition as routine	• Critically ill ICU patients with "very low" oral intake	1	6.2%
Focus on high protein intake (within oral route)	• "Due to muscle wasting"	2	12.5%

* Double coding applied (κ = 0.93). Each response was assigned to a macro-category; mentions of "protein" were collected as a transversal subtheme.

Question 18

"Why do you think oral intake decreases during hospitalization?"
(16 panelists – multiple reasons per response allowed)

Identified Macro-Cause*	Literal Examples	n panelists†	% (N = 16)
Poor palatability / presentation	"Food without good flavor," "unattractive diets," "limited variety"	11	68.8%
Clinical status / inflammation / disease	Anorexia due to acute illness, inflammatory process	11	68.8%
Cultural mismatch / dietary habits	"Diet does not fit their tastes/habits," "cultural relevance"	7	43.8%
Psychological factors	Stress, depression, mood	5	31.2%
Side effects of medications	—	2	12.5%
Logistical / service limitations	Small portion size, meal timing, "special situations"	2	12.5%
Frequent procedures / examinations	—	1	6.2%

Dysphagia and swallowing problems	—	1	6.2%

* Coding conducted by two independent reviewers (κ = 0.91).
† A single panelist could appear in multiple categories.

Question 19

"What alternatives do you propose to improve oral intake?"
(16 panelists; 1 = NA ⇒ N = 15 valid responses, multiple ideas per response)

Priority Strategy*	Literal Examples	n panelists	% (N = 15)
Improve presentation, taste, and variety of meals — hospital gastronomy, cyclic menus, "appealing" dishes, appropriate temperature	"Apply hospital gastronomy," "Improve the taste and appearance of the diet," "Presentation, variety of foods and preparations"	10	66.7%
Personalize/culturalize the diet — adjust to preferences, habits, and pathology	"Personalize the diet," "Variety and similarity to their usual diet," "Culturally and socially appropriate"	6	40.0%
Patient education and staff support — educational plan for patients, team training	"Educational plan… many patients don't even know what they are eating," "Support with intake by specific staff"	4	26.7%
Oral nutritional supplements (ONS)	"Offer oral supplements with acceptable organoleptic characteristics"	2	13.3%
Use of appetite stimulants / optimize medication	"Check with physician on the use of appetite stimulants"	2	13.3%
Increase frequency or portion size	"Small amounts more frequently," "Adjust portion size"	2	13.3%

* Thematic coding conducted by two reviewers (κ = 0.90). A single response could be included in more than one category; totals therefore exceed 100%.

Question 20

"How could unintentional weight loss be reduced at the hospital level?"
(16 panelists; one blank response ⇒ N = 15 analyzable)

Proposed Strategy*	Literal Examples	n	% (N = 15)
Early screening, monitoring, and timely intervention • initiation ≤ 48 h, risk detection, intake/tolerance monitoring, continuous adjustment	"Start management within the first 48 h with monitoring" · "Nutritional screening… timely intervention"	6	40%
Improve quality, density, and acceptability of hospital diet	"Better presentation of food" · "Adapting the diet to patient needs" · "Diet with high acceptability"	5	33%
Strengthen human resources and multidisciplinary approach • more dietitians, staff education, multimodal program with physiotherapy/psychology	"More staff dedicated to nutrition" · "Multimodal program involving nutrition, physical therapy, and psychology"	4	27%

Oral supplements / SA • specific additional intake		"Purchase of nutritional supplements supervised by nutrition" · "Supplements providing what is necessary"	2	13%

* Thematic coding performed by two reviewers (κ = 0.89). Responses could appear in multiple categories; therefore, percentages exceed 100%.

Question 24

"If the answer is yes, indicate how often you provide follow-up after discharge."
(16 panelists; 6 left the cell = NA ⇒ 10 interpretable responses)

Follow-up Category*	Classification Criterion	Literal Examples	n	% (N = 15†)
≤ 1 month (biweekly / monthly)	≤ 30 days, or "15 d then monthly"	"Monthly or quarterly"‡ · "Every 15 days" · "Generally monthly" · "Monthly" · "15 days and then monthly"	6	40%
2–3 months	31–90 days	"Once every 2 months" · "Every 3 months"	2	13%
> 3 months / late appointments	≥ 4 months or reports of delays > 90 d	"Appointments at COEX… 5 months after discharge"	1	7%
No follow-up / NA / no discharge / frequency not specified	Blank cell, "NA," "No discharge," "followed in outpatient clinic" (without periodicity)	—	6	40%

* Coding reached by consensus between two reviewers (κ = 0.88).
† Denominator = 15 panelists who confirmed providing follow-up in the filter question of round 1.
‡ Declared "Monthly or quarterly"; assigned to the ≤ 1 month subcategory to reflect the most frequently indicated option.

Question 25

"Would you like to clarify any of the questions you answered previously?"
(16 panelists – 9 responded "NA"; 7 useful contributions analyzed)

Clarification Theme*	Literal Examples	n
Educational continuity and outpatient follow-up	"Nutritional education is provided… and we request follow-up in outpatient clinic."	2
Description of internal flowcharts	"At HDT, screening is conducted between 7–8 a.m., we accompany the medical visit, and deliver a discharge plan—although not 100%."	1
Additional screening tools	"In addition to NRS we use MNA in older adults."	1
Structural limitations of the center	"UNAERC is an outpatient unit; there is no hospital food service, which is why some answers do not apply."	1
Heterogeneity of personal vs. hospital practice	"I combined what I do in clinic with data from discharged patients; I will check what the hospital does."	1
No comments / NA	—	9

* Thematic coding by consensus (κ = 0.86).

Supplementary Tables

Supplementary Table S1. Consolidated results of the 15 multiple-choice questions included in Round 1 of the Delphi study.

No.	Question	Option	Respondents (n / total)	Percentage (%)	Consensus
1	*Which screening tool do you use for nutritional risk?*	NRS	7 / 16	43.75	No
		MNA	3 / 16	18.75	No
		MST	2 / 16	12.50	No
		MUST	2 / 16	12.50	No
		Other	1 / 16	6.25	No
2	*How do you usually receive patients for nutritional assessment?*	Direct patient intake	11 / 16	68.75	No
		Paper-based referral	8 / 16	50.00	No
		Remote referral	5 / 16	31.25	No
3	*Which criteria or assessment method do you apply?*	GLIM criteria	11 / 16	68.75	No
		Subjective global assessment	7 / 16	43.75	No
		Objective assessment	7 / 16	43.75	No
		Other	1 / 16	6.25	No
4	*Does your hospital have a formal nutritional protocol?*	Yes	12 / 16	75.00	Yes
		No	4 / 16	25.00	No
7	*Do you perform systematic nutritional screening?*	Yes	14 / 16	87.50	Yes
		No	2 / 16	12.50	No
8	*Which method do you use to evaluate dietary intake?*	Usual diet	9 / 16	56.25	No
		24-hour recall	9 / 16	56.25	No
		Food frequency questionnaire	5 / 16	31.25	No
		No dietary assessment	1 / 16	6.25	No
9	*Do you apply a standardized nutritional evaluation form?*	No	9 / 16	56.25	No
		Yes	7 / 16	43.75	No
11	*How often do you monitor hospitalized patients' nutritional status?*	Once per week	7 / 16	43.75	No
		Monthly	6 / 16	37.50	No
		Other	3 / 16	18.75	No
		Every 15 days	0 / 16	0.00	No
12		Other	9 / 15	60.00	No
		Twice per week	4 / 15	26.67	No

	How often do you reassess nutritional risk in at-risk patients?	Once per week	1 / 15	6.67	No
		Monthly	1 / 15	6.67	No
		Every 15 days	0 / 15	0.00	No
14	*How frequently do you evaluate patients' dietary intake?*	Daily	6 / 16	37.50	No
		Other	4 / 16	25.00	No
		Once per week	3 / 16	18.75	No
		Three times per week	3 / 16	18.75	No
15	*Which anthropometric indicators do you routinely use?*	BMI	15 / 16	93.75	Yes
		Body composition	13 / 16	81.25	Yes
		Calf circumference	8 / 16	50.00	No
		Handgrip dynamometry	6 / 16	37.50	No
		Waist circumference	3 / 16	18.75	No
		Weight/Height (W/H)	2 / 16	12.50	No
		Skinfold thickness	1 / 16	6.25	No
16	*Which type of nutritional intervention do you prioritize?*	Optimization of oral diet	14 / 16	87.50	Yes
		Enteral nutrition	1 / 16	6.25	No
		Oral nutritional supplementation	1 / 16	6.25	No
		Mixed nutrition	0 / 16	0.00	No
		Parenteral nutrition	0 / 16	0.00	No
21	*Do you monitor patients after hospital discharge?*	Yes	14 / 16	87.50	Yes
		No	2 / 16	12.50	No
22	*How do you deliver nutritional education to patients?*	Written leaflet	6 / 14	42.86	No
		Verbal	6 / 14	42.86	No
		Detailed written report	2 / 14	14.29	No
23	*Do you provide continuity of nutritional care after discharge?*	Yes	10 / 16	62.50	No
		No	4 / 16	25.00	No
		Referral to health center	2 / 16	12.50	No

Each row presents the question number, response option, absolute frequency (n), relative frequency (%), and whether the pre-established consensus threshold (≥70%) was achieved. These findings summarize institutional practices, barriers, and strategies in hospital nutritional care, providing a structured foundation for subsequent Delphi rounds.

Supplementary Table S2. Results of Likert-Type Questions from Round 2 of the Delphi Consensus on Hospital Nutritional Care in Guatemala.

No.	Question	Response	n	Percent (%)	Agreement (%)	Consensus
1	*For adults <65 years, is NRS the best nutritional screening tool?*	Strongly agree	5/11	45.45	63.63	No
		Agree	2/11	18.18		
		Neither agree nor disagree	1/11	9.09		
		Disagree	2/11	18.18		
		Strongly disagree	1/11	9.09		
2	*For adults ≥65 years, is MNA the best nutritional screening tool?*	Strongly agree	5/11	45.45	81.81	Yes
		Agree	4/11	36.36		
		Disagree	1/11	9.09		
		Strongly disagree	1/11	9.09		
3	*Should the nutrition professional perform an objective nutritional assessment?*	Strongly agree	10/11	90.91	90.91	Yes
		Neither agree nor disagree	1/11	9.09		
4	*Should Subjective Global Assessment be completed with an objective evaluation?*	Strongly agree	9/11	81.82	81.82	Yes
		Neither agree nor disagree	2/11	18.18		
5	*If using GLIM criteria, should the evaluation be completed with an objective assessment?*	Strongly agree	8/11	72.73	90.91	Yes
		Agree	2/11	18.18		
		Neither agree nor disagree	1/11	9.09		
6	*Are medical history and baseline diagnosis part of the clinical assessment of nutritional status?*	Strongly agree	10/11	90.91	100	Yes
		Agree	1/11	9.09		
7	*Is review of drug–nutrient interactions part of the clinical assessment?*	Strongly agree	5/11	45.45	81.81	Yes
		Agree	4/11	36.36		
		Neither agree nor disagree	1/11	9.09		
		Disagree	1/11	9.09		

8	*Should lifestyle habits (alcohol, tobacco, drugs, contraceptives, exercise) be investigated?*	Strongly agree	8/11	72.73	100	Yes
		Agree	3/11	27.27		
9	*Should the physical exam include a systems review?*	Strongly agree	8/11	72.73	100	Yes
		Agree	3/11	27.27		
10	*In a detailed physical exam, should muscle mass, adipose tissue, and micronutrient deficiencies be evaluated?*	Strongly agree	8/11	72.73	100	Yes
		Agree	3/11	27.27		
11	*Is height determined by stadiometer, arm span, or knee height?*	Strongly agree	8/11	72.73	90.91	Yes
		Agree	2/11	18.18		
		Neither agree nor disagree	1/11	9.09		
12	*To estimate ideal weight, is the BMI formula used?*	Strongly agree	3/11	27.27	72.72	Yes
		Agree	5/11	45.45		
		Neither agree nor disagree	2/11	18.18		
		Strongly disagree	1/11	9.09		
13	*Is percentage weight loss an indicator of nutritional risk?*	Strongly agree	8/11	72.73	100	Yes
		Agree	3/11	27.27		
14	*Is BMI an indicator of nutritional status (deficit & excess)?*	Strongly agree	2/11	18.18	63.63	No
		Agree	5/11	45.45		
		Neither agree nor disagree	1/11	9.09		
		Disagree	1/11	9.09		
		Strongly disagree	2/11	18.18		
15	*Do BMI cutoffs vary for older adults?*	Strongly agree	6/11	54.55	90.91	Yes
		Agree	4/11	36.36		
		Neither agree nor disagree	1/11	9.09		

16	*Is bioimpedance feasible to assess body composition in hospitalized patients?*	Strongly agree<br>Agree<br>Neither agree nor disagree	6/11<br>4/11<br>1/11	54.55<br>36.36<br>9.09	90.91	Yes
17	*Is phase angle a raw, usable BIA value and prognostic indicator (disease, nutritional risk/status, function, mortality, length of stay)?*	Strongly agree<br>Agree<br>Neither agree nor disagree	6/11<br>4/11<br>1/11	54.55<br>36.36<br>9.09	90.91	Yes
18	*Is anthropometry an adequate method to assess body composition?*	Strongly agree<br>Agree<br>Neither agree nor disagree<br>Strongly disagree	3/11<br>6/11<br>1/11<br>1/11	27.27<br>54.55<br>9.09<br>9.09	81.82	Yes
19	*Does standardizing anthropometric measurements increase their reliability?*	Strongly agree<br>Agree	9/11<br>2/11	81.82<br>18.18	100	Yes
20	*Does WC ≥94 cm (men) and ≥80 cm (women) increase NCD risk?*	Strongly agree<br>Agree<br>Neither agree nor disagree	7/11<br>3/11<br>1/11	63.64<br>27.27<br>9.09	90.91	Yes
21	*In hospitalized adults, should sarcopenia be assessed?*	Strongly agree<br>Agree<br>Disagree	6/11<br>4/11<br>1/11	54.55<br>36.36<br>9.09	90.91	Yes
22	*Should muscle function be routinely assessed with handgrip dynamometry?*	Strongly agree<br>Agree<br>Neither agree nor disagree	5/11<br>4/11<br>2/11	45.45<br>36.36<br>18.18	81.81	Yes
23	*Is muscle strength an indicator of muscle mass, nutritional/health status, physical function, mortality, and LOS—thus to be used routinely?*	Strongly agree<br>Agree	5/11<br>6/11	45.45<br>54.55	100	Yes
24	*Calf circumference is an indirect measure of muscle mass; adjust for BMI/edema; routine*	Strongly agree<br>Agree	5/11<br>4/11	45.45<br>36.36	81.81	Yes

	cutoffs: Moderately Low: 34 cm men, 33 cm women; Severely Low: 32 cm men, 31 cm women.	Neither agree nor disagree	1/11	9.09		
		Strongly disagree	1/11	9.09		
25	*Should nutritional care protocols be updated every 2–5 years?*	Strongly agree	6/11	54.55	100	Yes
		Agree	5/11	45.45		
26	*Is a 24-h recall the right method for inpatient intake?*	Strongly agree	4/11	36.36	63.63	No
		Agree	3/11	27.27		
		Neither agree nor disagree	2/11	18.18		
		Disagree	1/11	9.09		
		Strongly disagree	1/11	9.09		
27	*Should a 5-point hedonic test be used to evaluate hospital diet acceptability?*	Strongly agree	7/11	63.64	90.91	Yes
		Agree	3/11	27.27		
		Neither agree nor disagree	1/11	9.09		
28	*Should anthropometric monitoring in hospitalized patients be done once per week?*	Strongly agree	4/11	36.36	81.81	Yes
		Agree	5/11	45.45		
		Disagree	2/11	18.18		
29	*Should anthropometric monitoring in outpatients be done every 1–3 months?*	Strongly agree	4/11	36.36	90.91	Yes
		Agree	6/11	54.55		
		Neither agree nor disagree	1/11	9.09		
30	*In critically ill inpatients, should biochemical monitoring be done every 24–48 h?*	Strongly agree	7/11	63.64	90.91	Yes
		Agree	3/11	27.27		
		Neither agree nor disagree	1/11	9.09		
31	*In inpatients on enteral/oral nutrition, should biochemical monitoring be done once per week?*	Strongly agree	5/11	45.45	72.72	Yes
		Agree	3/11	27.27		
		Strongly disagree	2/11	18.18		
		Disagree	1/11	9.09		
32	*In inpatients on parenteral nutrition, should biochemical monitoring be done every 24–72 h?*	Strongly agree	5/11	45.45	100	Yes
		Agree	6/11	54.55		
33		Strongly agree	5/11	45.45	100	Yes

	In outpatients, should biochemical monitoring be done every 1–3 months?	Agree	6/11	54.55		
34	*For renal pathology, are common labs: glucose, creatinine, BUN, electrolytes, uric acid, urine protein, urine glucose, CBC?*	Strongly agree<br>Agree	5/11<br>6/11	45.45<br>54.55	100	Yes
35	*For hepatic pathology, are common labs: glucose, creatinine, BUN, electrolytes, uric acid, triglycerides, total cholesterol, LDL, HDL, albumin, ALP, AST, ALT, total/direct/indirect bilirubin, CBC?*	Strongly agree<br>Agree	5/11<br>6/11	45.45<br>54.55	100	Yes
36	*For dyslipidemia, are common labs: glucose, creatinine, BUN, electrolytes, uric acid, triglycerides, total cholesterol, LDL, HDL, albumin, ALP, CBC?*	Strongly agree<br>Agree<br>Neither agree nor disagree	5/11<br>4/11<br>2/11	45.45<br>36.36<br>18.18	81.81	Yes
37	*In hospitalized patients, are common labs: glucose, creatinine, BUN, electrolytes, uric acid, triglycerides, total cholesterol, LDL, HDL, albumin, ALP, CRP, CBC?*	Strongly agree<br>Agree<br>Disagree	4/10<br>5/10<br>1/10	40<br>50<br>10	90	Yes
38	*In inpatients on parenteral nutrition, are common labs: glucose, creatinine, BUN, electrolytes, triglycerides, total cholesterol, LDL, HDL, albumin, AST, ALT, ALP, total/direct/indirect bilirubin, CBC?*	Strongly agree<br>Agree<br>Neither agree nor disagree	6/11<br>4/11<br>1/11	54.55<br>36.36<br>9.09	90.91	Yes
39	*Should inpatients receive daily clinical monitoring?*	Strongly agree<br>Agree<br>Neither agree nor disagree<br>Disagree	5/11<br>3/11<br>2/11<br>1/11	45.45<br>27.27<br>18.18<br>9.09	72.72	Yes
40	*Should inpatients receive clinical monitoring 3×/week?*	Strongly agree<br>Agree<br>Neither agree nor disagree<br>Strongly disagree	2/11<br>7/11<br>1/11<br>1/11	18.18<br>63.64<br>9.09<br>9.09	81.82	Yes

41	*Should oral diet be optimized whenever the GI tract is functional?*	Strongly agree<br>Agree	9/11<br>2/11	81.82<br>18.18	100	Yes
42	*Do you agree with using the decision-algorithm diagram for nutrition therapy?*	Strongly agree<br>Agree<br>Neither agree nor disagree	6/11<br>4/11<br>1/11	54.55<br>36.36<br>9.09	90.91	Yes
43	*Is oral intake reduced during hospitalization due to pathology-related factors?*	Strongly agree<br>Agree	9/11<br>2/11	81.82<br>18.18	100	Yes
44	*Is oral intake reduced during hospitalization due to organoleptic/cultural-relevance factors?*	Strongly agree<br>Agree	9/11<br>2/11	81.82<br>18.18	100	Yes
45	*Is oral intake reduced during hospitalization due to hospital-related factors (preparations/menu)?*	Strongly agree<br>Agree	8/11<br>3/11	72.73<br>27.27	100	Yes
46	*Is oral intake reduced during hospitalization due to restrictive diet modifications?*	Strongly agree<br>Agree	8/11<br>3/11	72.73<br>27.27	100	Yes
47	*Is oral intake reduced during hospitalization due to sociocultural factors?*	Strongly agree<br>Agree<br>Neither agree nor disagree	6/11<br>4/11<br>1/11	54.55<br>36.36<br>9.09	90.91	Yes
48	*Is oral intake reduced during hospitalization due to patient-related medical factors?*	Strongly agree<br>Agree<br>Neither agree nor disagree	6/11<br>4/11<br>1/11	54.55<br>36.36<br>9.09	90.91	Yes
49	*To improve oral intake: improve food preparation (hospital gastronomy, taste/appearance). Do you agree?*	Strongly agree<br>Agree	8/11<br>3/11	72.73<br>27.27	100	Yes
50	*To improve oral intake: multidisciplinary supervision of food/intake during stay. Do you agree?*	Strongly agree<br>Agree	9/11<br>2/11	81.82<br>18.18	100	Yes
51	*To improve oral intake: consider appetite stimulants. Do you agree?*	Strongly agree<br>Agree<br>Strongly disagree	6/11<br>4/11<br>1/11	54.55<br>36.36<br>9.09	90.91	Yes
52		Strongly agree	10/11	90.91	100	Yes

	Should patients receive nutritional follow-up after discharge?	Agree	1/11	9.09		
53	*After discharge, should nutritional monitoring occur at most every 3 months?*	Strongly agree<br>Agree<br>Disagree	7/11<br>3/11<br>1/11	63.64<br>27.27<br>9.09	90.91	Yes

Each row presents the item, the number of participants responding, the absolute and relative frequencies of favorable responses ("Agree" + "Strongly agree"), the calculated consensus percentage, and whether the pre-established threshold of 70% was reached. These findings reflect the structured evaluation of practices across the domains of nutritional screening, assessment, intervention, and monitoring.

Supplementary Table S3. Results of Likert-Type Questions from Round 3 of the Delphi Consensus on Hospital Nutritional Care in Guatemala

No.	Question	Response	n	Percent (%)	Agreement (%)	Consensus
1	*Is NRS the nutritional screening tool of choice for adults under 65 years?*	Strongly agree	8/12	66.67	100	Yes
		Agree	4/12	33.33		
2	*The method of estimating height using ulna length can be an alternative when measurement with stadiometer, arm span, or knee height is not possible.*	Strongly agree	5/12	41.67	100	Yes
		Agree	7/12	58.33		
3	*To estimate ideal weight, BMI can be used, individualizing cut-off points between 22–25 kg/m² depending on the patient's clinical condition.*	Strongly agree	6/12	50	100	Yes
		Agree	6/12	50		
4	*Percentage weight loss is an indicator of nutritional risk according to the attached cut-off points.*	Strongly agree	8/12	66.67	100	Yes
		Agree	4/12	33.33		
5	*BMI is a valid indicator of nutritional status, both deficit and excess, when combined with other indicators such as body composition, body fat percentage, and waist/hip ratio, among others.*	Strongly agree	7/12	58.33	100	Yes
		Agree	5/12	41.67		
6	*Handgrip measurement can be an alternative to assess muscle function when a dynamometer is not available.*	Strongly agree	4/12	33.33	100	Yes
		Agree	8/12	66.67		
7	*A 24-hour recall can be used to assess food intake during hospitalization.*	Strongly agree	6/12	50	83.33	Yes
		Agree	4/12	33.33		
		Neither agree nor disagree	1/12	8.33		
		Disagree	1/12	8.33		

8	*The plate photography method (before and after eating) can be an alternative to assess food intake during hospitalization.*	Strongly agree	4/12	33.33	91.67	Yes
		Agree	7/12	58.33		
		Strongly disagree	1/12	8.33		
9	*The usual diet can be used to assess food consumption at hospital admission.*	Strongly agree	5/12	41.67	100	Yes
		Agree	7/12	58.33		
10	*Anthropometric monitoring in hospitalized patients should be performed at least once a week.*	Strongly agree	6/12	50	100	Yes
		Agree	6/12	50		
11	*Biochemical monitoring in hospitalized patients with enteral/oral nutrition should be performed at least once a week.*	Strongly agree	7/12	58.33	100	Yes
		Agree	5/12	41.67		

For each item, the number of participants responding, the absolute and relative frequencies of favorable responses (“Agree” + “Strongly agree”), the calculated consensus percentage, and the classification of consensus achieved (Yes/No) are presented. All 11 items reached the predefined threshold of ≥70%, with several achieving 100% agreement, confirming the robustness of the final set of recommendations.

Supplementary Table S4. Alignment of Delphi Round Questions with Final Recommendations for Hospital Nutrition Care in Guatemala

Question(s)	Round	Consensus achieved	Recommendation	General Domain
R1–Q1: "Which screening tool do you use for nutritional risk?" (NRS, MNA, MST, MUST, other). R2–Item: "For adults <65 years, NRS should be the preferred screening tool." R2–Item: "For adults ≥65 years, MNA should be the preferred screening tool." R3–Item: "Confirm NRS as the preferred tool for adults <65 years."	R1; R2; R3	R1: No consensus (heterogeneous use).<br>R2: MNA ≥65: Consensus (≥70%).<br>R3: NRS <65: Consensus (100%).	Use the NRS in adults under 65 years and the MNA in adults over 65 years as standardized nutritional screening tools.	Screening
R1–Q3 (open): "Which criteria do you use for clinical nutritional assessment? (GLIM, SGA, other)." R2–Q3–Q8 (Likert statements): "Every hospitalized patient should undergo an objective nutritional assessment upon admission"; "SGA should be complemented with objective measures"; "GLIM criteria must include muscle mass and biochemical parameters"; "Relevant clinical history and medical diagnoses should be included in the assessment"; "Drug–nutrient interactions should be systematically investigated"; "Lifestyle habits (alcohol, tobacco, drugs, physical activity, contraceptives) must be systematically inquired." R3 (various items): Confirmed agreement on objective assessment, GLIM criteria, and incorporation of habits and interactions.	R1; R2; R3	R1: No consensus, heterogeneity in methods.<br>R2: High consensus (≥90%) for objective assessment, SGA + objective measures, GLIM + objective parameters, clinical history, drug–nutrient interactions, and lifestyle habits.<br>R3: All reaffirmed with consensus (≥90–100%).	Conduct an objective nutritional assessment for every hospitalized patient upon admission.<br>Complement the SGA with objective measurements to achieve a comprehensive nutritional evaluation.<br>When applying GLIM criteria, include objective measures such as muscle mass and biochemical parameters.<br>Incorporate relevant clinical history and medical diagnoses as part of the nutritional evaluation.<br>Investigate and document potential drug–nutrient interactions during the clinical assessment.<br>Systematically inquire about lifestyle habits with possible nutritional impact: alcohol, tobacco, drug use, physical activity, and contraceptives.	Clinical assessment
R2–Q9: "A systems-based review should be included as part of the nutritional physical examination." R2–Q10: "Muscle mass, adipose tissue, and clinical signs of micronutrient deficiencies should be routinely assessed."	R2	Q9 and Q10: Full consensus (100%). Both items unanimously validated as standard components of nutritional physical examination.	Include a systems-based review as part of the nutritional physical examination.<br>Routinely assess muscle mass, adipose tissue, and clinical signs of micronutrient deficiencies.	Physical examination

R2–Q11: "Height should be measured using a stadiometer, arm span, or knee height." R3–Q2: "When other tools are unavailable, ulna length should be used to estimate height." R2–Q12: "Ideal weight should be estimated using a reference BMI of 22–25 kg/m², adjusted to clinical condition." R2–Q13: "For adults >65 years, physiological BMI variations should be considered for proper interpretation." R2–Q14: "Percentage of weight loss should be interpreted as a clinical indicator of nutritional risk." R3–Q4 & Q7: "BMI interpretation should only be validated when combined with body composition and central adiposity measures."	R2; R3	R2: Consensus on height measurement, BMI reference 22–25, older adult variations, and % weight loss as risk (≥81.8%).<br>R3: Consensus on ulna length as alternative (100%) and on contextualized BMI use with body composition (100%).	Measure height using a stadiometer, arm span, or knee height; use ulna length when other methods are unavailable.<br>Estimate ideal weight using a reference BMI of 22–25 kg/m², adjusted to the patient's clinical condition.<br>Consider physiological variations of BMI in adults over 65 years for adequate interpretation.<br>Interpret percentage weight loss as a clinical indicator of nutritional risk.<br>Validate the use of BMI only when combined with body composition and central adiposity measures.	Anthropometry
R1–Q15: "Clinical anthropometry methods (including handgrip, calf circumference, waist circumference, etc.)." R2–Q16: "Use of bioelectrical impedance analysis (BIA) in hospitalized patients." R2–Q17: "Interpretation of phase angle (PhA) derived from BIA as a prognostic parameter." R2–Q18–Q20: "Use of anthropometry as complementary method; standardization of measurement procedures." R2–Q21: "Waist circumference cut-offs ≥94 cm (men) and ≥80 cm (women) as NCD risk indicators." R2–Q22: "Assessment of sarcopenia risk in adults (clinical/instrumental)." R2–Q23–Q24: "Measurement of calf circumference with cut-offs 34/33 cm (moderate) and 32/31 cm (severe)." R3–Q8: "Confirmation of calf circumference as indicator of muscle mass."	R1; R3	R2: Consensus achieved for BIA (90%), PhA (90%), anthropometry as complementary (≥90%), waist circumference cut-offs (90.9%), sarcopenia assessment (90.9%), calf circumference cut-offs (81.8%).<br>R3: Confirmatory consensus on calf circumference (81.8%).	Use bioelectrical impedance analysis (BIA) in hospitalized patients as a feasible tool for body composition assessment.<br>Interpret the phase angle derived from BIA as a nutritional prognostic parameter.<br>Recognize the utility of clinical anthropometry as a complementary method in body composition assessment.<br>Standardize procedures for anthropometric measurements in the hospital setting.<br>Consider waist circumference cut-offs ≥94 cm in men and ≥80 cm in women as indicators of high risk for chronic noncommunicable diseases.<br>Assess sarcopenia risk in hospitalized adults using clinical or instrumental methods.<br>Measure calf circumference as an indirect indicator of muscle mass, using cut-offs of 34/33 cm (moderate risk) and 32/31 cm (severe risk).	Body composition
R1–Q15 (part): Question on anthropometric methods, which included handgrip dynamometry as part of the evaluation. R2–Q22: "Routine measurement of muscle strength using handgrip dynamometry." R2–Q23: "Use of handgrip strength as a valid alternative when a dynamometer is not available."	R1; R2	R1: Preliminary identification of handgrip as part of institutional practices (heterogeneous, no consensus).<br>R2: Full consensus: handgrip as a routine measure of muscle strength (100%); consensus on using handgrip as a valid alternative if a dynamometer is unavailable (100%).	Routinely measure muscle strength using handgrip dynamometry.<br>Use handgrip strength as a valid clinical alternative when a dynamometer is not available.	Functionality

R1–Q5 (open): "How often are institutional protocols on nutritional care updated?" (answers ranged from every 1 to 5 years). R2–Q25: "Institutional protocols on hospital nutritional care should be updated every 2 to 5 years."	R1; R2	R1: Heterogeneous responses (updates ranged 1–5 years; no consensus reached).<br>R2: Full consensus (100%) on updating protocols every 2–5 years.	Update institutional protocols on hospital nutritional care every 2 to 5 years.	Quality management
R1–Q10 (open): "How is hospital diet acceptability assessed in your institution?" (open responses included patient surveys, informal feedback, and rarely structured tools). R2–Q27: "Implement 5-point hedonic scales to evaluate hospital diet acceptability."	R1; R2	R1: High heterogeneity; no consensus on standardized tools (methods varied across hospitals).<br>R2: Consensus reached (90.9%) supporting the use of a 5-point hedonic scale as the standardized approach.	Implement 5-point hedonic scales to evaluate hospital diet acceptability.	Diet acceptability
R1–Q11: "How often are anthropometric measurements performed in hospitalized patients?" (open-ended; responses varied from admission-only to irregular follow-up). R2–Q28: "Perform weekly anthropometric measurements in hospitalized patients."	R1; R2	R1: Responses revealed wide variability with no consensus (frequency ranged from admission-only to biweekly).<br>R2: Consensus achieved (81.8%) to establish weekly anthropometric monitoring in hospitalized patients.	Perform weekly anthropometric measurements in hospitalized patients.	Inpatient monitoring
R1–Q24: "How is nutritional follow-up performed in outpatients?" (open-ended; responses varied from sporadic to every 6 months). R2–Q29: "Perform anthropometric measurements in outpatients every 1 to 3 months, depending on nutritional risk." R3–Q11: "Outpatients at nutritional risk should undergo anthropometric monitoring at least every 3 months."	R1; R3	R1: No consensus (practices varied widely across institutions).<br>R2: Consensus achieved (90.9%) on 1–3 month monitoring intervals.<br>R3: Reinforcement with strong consensus (100%) confirming structured follow-up every ≤3 months.	Perform anthropometric measurements in outpatients every 1 to 3 months, depending on nutritional risk.	Outpatient monitoring
R2–Q30: "ICU patients should undergo biochemical monitoring every 24–48 hours."	R2	Consensus achieved (≈90.9%) supporting 24–48 h monitoring in ICU settings.	Monitor biochemical parameters every 24–48 hours in ICU patients.	Critical care biochemistry
R2–Q31: "Hospitalized patients on enteral and/or oral nutritional support should undergo biochemical monitoring at least once per week."	R2	Consensus achieved (≈81.8%) confirming weekly monitoring.	Monitor biochemical parameters at least once per week in patients receiving enteral and/or oral nutritional support.	Inpatient biochemistry
R2–Q32: "Patients receiving parenteral nutrition should undergo biochemical monitoring every 24–72 hours."	R2	Consensus achieved (≈90.9%) supporting 24–72 h monitoring for PN.	Monitor biochemical parameters every 24–72 hours in patients receiving parenteral nutrition.	Inpatient biochemistry
R2–Q33: "Outpatients should undergo biochemical monitoring every 1–3 months."	R2	Consensus achieved (100%) confirming 1–3 month intervals for outpatient biochemical monitoring.	Schedule biochemical monitoring in outpatients every 1 to 3 months.	Outpatient biochemistry

R1–Q13 (open-ended): "Which biochemical parameters are routinely monitored in hospitalized patients?"	R1	Responses heterogeneous; used to inform structured R2 items.	Establish standardized biochemical panels differentiated by pathology.	Biochemical parameters
R2–Q30–Q38: Panels for renal, hepatic, dyslipidemia, PN, and general inpatient evaluation.	R2	Consensus achieved (100%) for pathology-specific biochemical panels (renal, hepatic, dyslipidemia, parenteral nutrition, general hospitalized patients).	In patients with liver disease: glucose, creatinine, BUN, electrolytes, uric acid, triglycerides, total cholesterol, LDL, HDL, albumin, alkaline phosphatase, AST, ALT, total/direct/indirect bilirubin, and CBC.<br>In patients with kidney disease: glucose, creatinine, BUN, electrolytes, uric acid, urine protein and glucose, and CBC.<br>In patients with dyslipidemia: glucose, creatinine, BUN, electrolytes, uric acid, triglycerides, total cholesterol, LDL, HDL, albumin, alkaline phosphatase, and CBC.<br>In hospitalized patients: glucose, creatinine, BUN, electrolytes, uric acid, triglycerides, total cholesterol, LDL, HDL, albumin, alkaline phosphatase, CRP, and CBC.<br>In patients with PN: glucose, creatinine, BUN, electrolytes, triglycerides, total cholesterol, LDL, HDL, albumin, AST, ALT, alkaline phosphatase, total/direct/indirect bilirubin, and CBC.	Biochemical parameters
R2–Q39–Q40: Statements on frequency of clinical monitoring (daily or at least three times per week in hospitalized patients).	R2	Consensus achieved (≥80%) – panel agreed on systematic clinical monitoring as part of routine nutritional follow-up.	Record daily clinical monitoring as part of nutritional follow-up.<br>Record clinical monitoring at least three times per week in hospitalized patients.	Clinical monitoring
R1–Q17 (open-ended): Practices for optimizing oral diet and triggers for escalation to other nutritional support.<br>R2–Q41–Q42: Likert-type statements on optimizing oral diet whenever the gastrointestinal tract is functional and applying structured decision-making algorithms.	R1; R2	Consensus achieved in R2 (100%) – unanimous agreement on prioritizing oral diet and using algorithms to guide clinical decisions.<br>R1 responses reflected heterogeneity and local practices without clear consensus.	Optimize oral diet whenever the gastrointestinal tract is functional.<br>Apply clinical decision-making algorithms for selecting the most appropriate nutritional therapy (Figure 2).	Dietary intervention

R1–Q18 (open-ended): Exploration of barriers to hospital food intake (medical, sensory, cultural, social). R2–Q43–Q48: Likert-type statements detailing the multifactorial causes of reduced intake, including medical conditions, organoleptic properties of food, cultural practices, and social factors.	R1; R2	Consensus achieved in R2 (100%) – full agreement that barriers to intake are multifactorial.<br>R1 responses highlighted variability but confirmed the multidimensional nature of barriers.	Recognize the multifactorial causes of reduced intake, including medical, organoleptic, cultural, and social factors.	Barriers to intake
R1–Q19 (open-ended): Strategies suggested by experts to improve food preparation, presentation, and variety in hospitals. R2–Q49–Q51: Likert-type statements on (a) improving preparation/presentation/variety, (b) ensuring multidisciplinary support to monitor intake, and (c) considering appetite stimulants when intake is reduced without reversible cause.	R1; R2	Consensus achieved in R2 (≥90% across all three items).<br>R1 responses provided heterogeneous strategies, but R2 structured statements reached strong agreement.	Implement strategies to improve the preparation, presentation, and variety of hospital foods.<br>Ensure multidisciplinary support in monitoring food intake.<br>Consider the use of appetite stimulants in cases of decreased intake without reversible cause.	Intake improvement
R1-Q8 ("Which dietary assessment methods are applied at admission and during follow-up?") R2-Q26 ("Use of 24-hour recall and photographic methods to evaluate hospital diet") R3-Q7 ("Consolidated recommendation: usual diet at admission, 24-hour recall, photographic plate methods")	R1; R3	R1: No consensus (heterogeneous answers)<br>R2: 63.6% (below 70%, no consensus)<br>R3: consensus (Agree + Strongly Agree)	Record the patient's usual diet at admission as a baseline for nutritional follow-up.<br>Use the 24-hour recall as a valid tool to estimate hospital intake in clinically stable patients.<br>Employ photographic plate methods before and after meals as a practical visual alternative to assess hospital food consumption.	Dietary Evaluation
R1–Q24 (open-ended, continuity of care): Experts described institutional practices and challenges for outpatient follow-up, including lack of standardized schedules. R1–Q25 (open-ended, barriers): Mentioned variability in access to outpatient services. R2–Q52: Likert-type statement: "Hospitalized patients should receive structured nutritional follow-up after discharge." R2–Q53: Likert-type statement: "Nutritional status should be monitored at least every three months during follow-up."	R1; R2	Consensus achieved in Round 2 (100%) for both Q52 and Q53.<br>R1 highlighted heterogeneity and lack of standardization, which motivated formal statements in R2.	Provide structured nutritional follow-up after hospital discharge.<br>Monitor patients' nutritional status at least every three months during post-discharge follow-up.	Post-discharge follow-up
